# Ubiquitin-associated protein 2 like (UBAP2L) enhances growth and metastasis of gastric cancer cells

**DOI:** 10.1080/21655979.2021.1982308

**Published:** 2021-11-25

**Authors:** Sihan Lin, Zhiyong Yan, Qiaofei Tang, Shuang Zhang

**Affiliations:** a Department of Surgical Oncology, The First Hospital of China Medical University, Shenyang, People’s Republic of China; b Department of Otorhinolaryngology, The Second Affiliated Hospital of Shenyang Medical College, Shenyang, People’s Republic of China

**Keywords:** UBAP2L, gastric cancer, miRNA, growth, migration, invasion

## Abstract

Ubiquitin-proteasome pathway has emerged as therapeutic targets for cancer. GEPIA database analysis showed that the expression of ubiquitin-associated protein 2 like (UBAP2L) in gastric cancer specimens was significantly higher than that in non-tumor tissue, and its high expression is associated with poor survival of gastric cancer patients. This study aims to investigate the role of UBAP2L in gastric cancer. Real-time PCR and western blot results showed that UBAP2L expression was upregulated in gastric cancer cell lines. Loss- and gain-of-function experiments demonstrated that silencing of UBAP2L inhibited proliferation, migration and invasion, and induced apoptosis of gastric cancer cells, and overexpression of UBAP2L played opposite roles. Nude mice inoculated with UBAP2L-silenced gastric cancer cells generated smaller xenografted tumors *in vivo*. Furthermore, UBAP2L activated Wnt/β-catenin signaling – the accumulation of nuclear β-catenin and the expression of its downstream targets (cyclin D1, AXIN-2 and c-MYC) was facilitated, whereas knockdown of UBAP2L deactivated this signaling. The tumor-suppressing effect of UBAP2L silencing was abolished by forced activation of β-catenin^S33A^. UBAP2L has been confirmed as a novel and direct target of miR-148b-3p. The anti-tumor effect of miR-148b-3p was partly reversed by UBAP2L overexpression. The expression of miR-148b-3p was negatively correlated with that of UBAP2L in gastric cancer samples. Overall, our study indicates that UBAP2L is required to maintain malignant behavior of gastric cancer cells, which involves the activation of Wnt/β-catenin signaling pathway. We propose UBAP2L as a potential therapeutic target against gastric cancer.

## 1. Introduction

As one of the most common cancers, gastric cancer is a leading cause of cancer death all over the world [1]. In 2012, there were about 951 thousand new gastric cancer cases and 723 thousand deaths [2]. The pathogenesis of gastric cancer is linked with multiple risk factors, including *Helicobacter pylori* infection, diet, smoking, drinking and familial inheritance [2,3]. Although there has been a decline in the morbidity and mortality of gastric cancer due to the improvement of diet in recent years, the burden of the disease remains high worldwide [1]. Gastrectomy is considered as the only sanative therapeutic method for gastric cancer patients, but it may not be feasible for patients with distal metastasis [1]. The overall survival rate of patients with advanced or metastatic gastric cancer is less than 12 months [1]. Some biological molecules are considered as diagnostic markers or therapeutic targets for human gastric cancer. For instance, *HER2* gene is reported to be involved in the development of gastric cancer. About 12–20% of the gastric adenocarcinomas are HER2 positive (with gene amplification and/or ectopic expression of protein) [4]. Combination treatment of chemotherapy with trastuzumab, a HER2-specific antibody, could lengthen overall survival and progression-free survival in HER2-positive gastric cancer patients [5].

The ubiquitin-proteasome pathway is generally responsible for protein degradation in vertebrate and controls multiple life events [6,7]. Inhibition of ubiquitin-proteasome pathway is considered as an efficient therapeutic approach to cancer, and several inhibitors of proteasome have been approved for use in clinical treatment of cancer [8–11]. Ubiquitin-associated protein 2-like (UBAP2L) is an evolutionarily conserved protein, which possesses an N-terminal ubiquitin-associated domain. UBAP2L is found to aggregate after treatment with a proteasome inhibitor *in vitro* [12], suggesting that it is likely to be involved in the ubiquitin-proteasome pathway. UBAP2L has been reported to be highly expressed in multiple cancers, and knockdown of UBAP2L could suppress malignant behaviors in cancer cells, including breast cancer, hepatocellular carcinoma, and prostate cancer [13–15]. However, the function of UBAP2L in gastric cancer awaits further investigation.

UBAP2L expression is reported to be increased in gastric cancer tissues in the database, and our data demonstrate its elevation in gastric cancer specimens and cell lines. The gain- and loss-of-function study of UBAP2L was performed in gastric cancer cells by using overexpression plasmids and small interfering RNA. In addition, UBAP2L was confirmed to be a potential gene targeted by miR-148b-3p. MiR-148b-3p is a deeply conserved microRNA with tumor-suppressing function. MiR-148b-3p was reported to suppress metastasis of gastric cancer cells *in vitro* and *in vivo* [16]. Ding *et al*. demonstrated that miR-148b-3p inhibited gastric cancer cell growth and invasion by blocking glycolysis [17]. Song *et al*. reported that miR-148b-3p was decreased in gastric cancer tissues, and that overexpression of miR-148b-3p restrained the growth of gastric cancer cells both *in vivo* and *in vitro* [18]. Bioinformatic predictions indicate that UBAP2L might be a target of miR-148b-3p.

Based on findings previously described, we speculated that UBAP2L promoted malignancies of gastric cancer cells by activating Wnt/β-catenin signaling, and its expression was regulated by miR-148b-3p. The present study aims to investigate the roles of UBAP2L in gastric cancer cells and the regulation of miR-148b-3p on UBAP2L expression via experiments *in vivo* and *in vitro*.

## 2. Material and methods

### 2.1. Clinical specimens

Twenty-seven pairs of clinical gastric cancer and para-carcinoma samples were collected from the patients who underwent gastrectomy at the First Hospital of China Medical University. Patients received neither chemotherapy nor radiotherapy before surgical resection. Gastric cancer was diagnosed by pathological staining. The paired para-carcinoma tissue was isolated from the normal gastric mucosa of the same patient (≥5 cm away from cancer lesion). The informed consent was obtained from each patient. All experiments on human specimens were performed according to the Declaration of Helsinki, and approved by the ethics committee of The First Hospital of China Medical University with approval number [2019]110.

### 2.2. Construction of plasmid

To construct the UBAP2L overexpression plasmid, the open reading frame (ORF) sequence of human UBAP2L was cloned into *HindIII* and *XhoI* sites of pcDNA3.1 vector.

For silencing of UBAP2L, the siRNA sequence targeting UBAP2L ORF sequence was loaded into pRNAH1.1 vector with *BamHI* and *HindIII* restriction enzyme sites.

The UBAP2L 3ʹUTR sequence containing the binding sites for miR-148b-3p and the mutant sequence were synthesized, and ligated into the pmirGLO vector with restriction enzymes *NheI* and *XhoI*.

### 2.3. Cell culture and transfection

Normal human gastric mucosa cell line (GES-1), and gastric cancer cell lines (MKN-45, Hs746T, and NCI-N87) were purchased from FUHENG (Beijing, China), and gastric cancer cell line AGS from ZQXZ (Shanghai, China). The AGS cells were cultured with F12K medium, MKN-45 and NCI-N87 with RPMI-1640 medium and GES-1 and Hs726T cells with DMEM medium with 10% fetal bovine serum (FBS) in an incubator with 37°C under 5% CO_2_.

AGS, NCI-N87, MKN-45 and Hs746T cells in a serum-free medium were transfected with miRNA mimics/siRNA/plasmid using lipofectamine 2000 reagent (Invitrogen, Carlsbad, USA).

For stable silencing UBAP2L, pRNAH1.1 vector containing shUBAP2L sequence or negative control (NC) was transfected into AGS and NCI-N87 cells. Forty-eight hours later, the cells were treated with G418 (400 μg/ml for AGS cells and 300 μg/ml for NCI-N87 cells) for 6 weeks until the monoclonal cell masses appeared. The cells stably silencing UBAP2L were cultured for subsequent experiments.

For the enhanced expression of UBAP2L, the UBAP2L overexpression plasmid, or pcDNA3.1 empty vector was transfected into MKN-45 and Hs746T cells.

To continuously express β-catenin, the overexpression plasmid generated from wild-type β-catenin with mutation of Ser33 to Tyr in pcDNA3.1 vector was transfected into UBAP2L-silenced AGS cells.

### 2.4. Real-time PCR

Real-time PCR was used for detection of mRNA level of UBAP2L and expression of miR-148b-3p.

Total RNA was extracted using an RNApure kit, and the sample concentration was measured with a spectrophotometer. cDNA was obtained via reverse transcription, and used for real-time quantitative PCR to determine the levels of UBAP2L and miR-148b-3p. GAPDH served as an internal control for UBAP2L, and U6 for miR-148b-3p. The data were analyzed using 2^−ΔΔCt^ method [19]. The primer sequence is shown in Table 1.

**Table 1.** Sequence information on primers, siRNA and mimics/inhibitor in this study

Name	Sequence (5ʹ-3ʹ)
siUBAP2L-1 sence	GCGGCAAUACGUGGAACAATT
siUBAP2L-1 antisence	UUGUUCCACGUAUUGCCGCTT
siUBAP2L-2 sence	GCUAAAGGCGGCAGUACUATT
siUBAP2L-2 antisence	UAGUACUGCCGCCUUUAGCTT
siRNA NC sence	UUCUCCGAACGUGUCACGUTT
siRNA NC antisence	ACGUGACACGUUCGGAGAATT
miR-148-3p mimics	UCAGUGCAUCACAGAACUUUGU
	AAAGUUCUGUGAUGCACUGAUU
mimics NC	UUCUCCGAACGUGUCACGUTT
	ACGUGACACGUUCGGAGAATT
miR-148-3p inhibitor	AGUGAAUUCUACCAGUGCCAUA
inhibitor NC	UUGUACUACACAAAAGUACUG
UBAP2L F	ATTCCTGGGAGATGGTCG
UBAP2L R	CCTTCTGCCTCTTTCTGTTC
GAPDH F	GACCTGACCTGCCGTCTAG
GAPDH R	AGGAGTGGGTGTCGCTGT
miR-148b-3p RT	GTTGGCTCTGGTGCAGGGTCCGAGGTATTCGCACCAGAGCCAACACAAAG
miR-148-3p F	TCAGTGCATCACAGAACTTTGT
miR-148-3p R	GCAGGGTCCGAGGTATTC
U6 RT	GTTGGCTCTG GTGCAGGGTCCGAGGTATTCGCACCAGAGCCAACAAAATATGG
U6 F	GCTTCGGCAGCACATATACT
U6 R	GCAGGGTCCGAGGTATTC

### 2.5. Western blot

Western blots were performed to measure the expression levels of proteins [20].

Total protein was extracted using RIPA buffer, and cytoplasmic and nuclear fractions were separated with a cytoplasmic and nuclear protein extraction kit (Solarbio). SDS-PAGE was applied to separate proteins of different sizes, and the proteins were transferred onto PVDF membranes. Skim milk was used to block the nonspecific antigens, and then the membrane carrying proteins was incubated with antibodies against following proteins: UBAP2L (1:1000; Affinity, Changzhou, China), cyclin D1 (1:1000; Abclonal, Wuhan, China), AXIN2 (1:1000; Affinity), c-MYC (1:1000; Affinity), β-catenin (1:1000; Abclonal), GAPDH (1:10,000; Proteintech, Wuhan, China) or histone H3 (1:5000; GeneTex, Irvine, USA). After rinsing the membrane to remove unbound primary antibodies, the membrane was incubated with a secondary antibody conjugated with HRP (1:3000; Solarbio) for 1 h at 37°C. Subsequently, the protein reacted with ECL reagents, and signal exposure was carried out in the dark. GAPDH served as the cellular or cytoplasmic internal control, and histone H3 as the nuclear internal control.

The primer information was shown in Table 1.

### 2.6. CCK-8 assay

The cell viability was measured with a cell counting kit (CCK)-8 [21]. After cell culture in 96-well plates for 0–72 hours, CCK-8 reagent (KeyGen, Nanjing, China) was added at 10 μl per well to treat cells for 1 h. The optical density at 450 nm was then determined with a microplate reader.

### 2.7. Flow cytometry

Flow cytometry was carried out to measure the cell apoptosis and cell cycle [22].

For apoptosis detection, the collected cells were treated with an Annexin V-FITC detection kit (Beyotime, Haimen, China) in the dark, and analyzed with the flow cytometer (ACEA, San Diego, USA).

To detect cell cycles, the collected cells were washed with PBS, and incubated with PI/RNaseA reagents (Beyotime) in the dark. The percentage of cells in each phase of the cell cycle was analyzed with the flow cytometer (ACEA). Proliferation index (PI) was calculated as follows: PI = (G2/M + S)/G1/G0.

### 2.8. Wound healing

Wound healing assay was performed to evaluate cell migratory ability [23]. The cells were pre-treated with mitomycin C (Sigma, St. Louis, USA) in a serum-free medium for 1 h. A 200-μl pipette tip was used to make a scratch and the cells were continued to culture for 24 h. The wound size was measured based on the wound photograph at 100× magnification, and migration rate was calculated.

### 2.9. Transwell assay

The cell invasive ability was measured by transwell assay [24]. Matrigel (BD, Franklin Lakes, USA) was pre-paved on the polycarbonate membrane to mimic the extracellular matrix. The cells were seeded in upper chambers without serum, and the medium containing 30% FBS was added to the lower chambers. After the culture for 24 h, the chamber was taken out, and the cells on the reverse surface of the polycarbonate membrane were stained with 0.5% crystal violet (Amresco, Solon, USA) and counted.

### 2.10. Immunofluorescent staining

Immunofluorescent staining was carried out to determine the expression and distribution of β-catenin in gastric cancer cells. The detailed steps referred to the previously reported [25]. Briefly, the cells cultured on the glass slide were fixed with 4% paraformaldehyde (Sinopharm, Beijing, China) and subjected to 0.1% Triton X-100 to permeate cytomembrane. The cells were then blocked with goat serum for 30 min and incubated with primary antibody against β-catenin (1:50; Abclonal) at 4°C overnight. The unbound antibodies were washed off with PBS, and secondary antibodies conjugated with Cy3 (1:200; Beyotime) were applied to react with primary antibodies. Nuclei were stained with DAPI, and antifading reagent (Solarbio) was used to prevent fluorescence attenuation. Finally, the slides were observed with a fluorescence microscope.

### 2.11. Xenograft model

Xenograft experiment was performed to measure the tumorigenic ability of gastric cancer cells [26]. The animals used in this study were fed according to the Guide for the Care and Use of Laboratory Animals (8th). The procedure of animal experiment was approved by the ethic committee of Shenyang Medical College.

Male BALB/c nude mice were grouped randomly into four groups: AGS-shRNA NC, AGS-shUBAP2L, NCI-N87-shRNA NC, NCI-N87-shUBAP2L. The AGS or NCI-N87 cells stably transfected with shRNA NC or shUBAP2L were subcutaneously injected into the mice (5 × 10^6^ cells each mouse). The size of subcutaneous nodules was measured every 3 days. Nineteen days after injection, the mice were sacrificed, and the nodules were separated for subsequent detections.

### 2.12. Immunohistochemical staining

Immunohistochemical staining was used for the determination of Ki-67 expression in subcutaneous tumors from animals [27]. The tumor tissues were fixed with formaldehyde, dehydrated with ethanol and xylene, and embedded in paraffin and sectioned into 5-μm thickness. Subsequently, the sections underwent deparaffinization and antigens retrieval by boiling. After blocking with goat serum, and the sections were incubated with primary antibody against Ki-67 (1:200; Abclonal) at 4°C overnight and then HRP-conjugated secondary antibodies (1:500; Thermo Scientific, Waltham, USA). The sections reacted with diaminobenzidine (DAB) reagent (Solarbio) for several seconds, and the nuclei were counterstained with hematoxylin (Solarbio). Finally, the tissue sections were mounted with gum, and photographed.

### 2.13. Dual-luciferase reporter assay

The binding between miR-148b-3p and UBAP2L was predicted using bioinformatic website TargetScan (http://www.targetscan.org/vert_71/), and dual-luciferase reporter assay was used to confirm this binding [28]. The pmirGLO vector containing UBAP2L 3ʹUTR or its mutant sequence was co-transfected into 293 T cells with miR-148b-3p mimics. Forty-eight hours later, a dual-luciferase reporter gene assay kit (KeyGen) was used to measure the luciferase activity.

### 2.14. Statistical analysis

In this study, the data were presented as mean ± SD. The data from clinical specimens were analyzed with *Student’s t* test. The correlation between UBAP2L and miR-148b-3p were analyzed with the Spearman test. The data from cell experiments from three individuals, and data from animal experiments from six individuals were analyzed with ANOVA test with post *hoc Bonferroni’s* multiple comparisons. *p* value less than 0.05 were regarded as statistically significant.

## 3. Results

Herein, to investigate the roles of UBAP2L and miR-148b-3p in gastric cancer, we detected their expression in clinical gastric cancer specimens and changed their expression in gastric cancer cells. We found that UBAP2L was increased in gastric cancer specimens, and its expression was negatively correlated with that of miR-148b-3p. UBAP2L promoted proliferation, migration, invasion and tumorigenesis, and suppressed apoptosis of gastric cancer cells. UBAP2L activated Wnt/β-catenin signaling, and the alterations induced by UBAP2L knockdown were reversed by continuous expression of β-catenin. In addition, UBAP2L was confirmed to be a target of miR-148b-3p, and the tumor-suppressing effect of miR-148b-3p was antagonized by UBAP2L.

### 3.1. The expression of UBAP2L was increased in gastric cancer specimens

**Figure 1.**
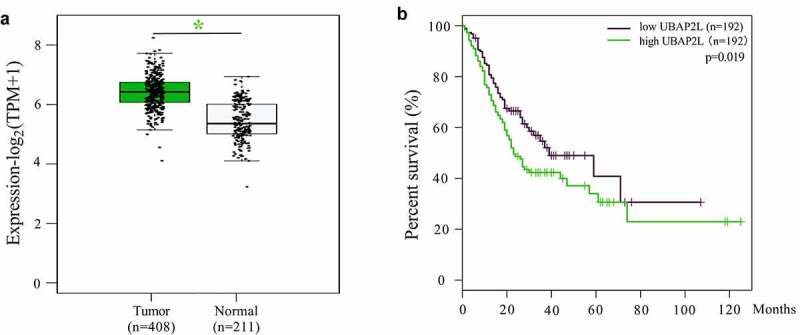
The expression of UBAP2L in gastric cancer and survival of gastric cancer patients from GEPIA database

According to the Gene Expression Profiling Interactive Analysis (GEPIA) database, UBAP2L mRNA levels were markedly higher in gastric cancer tissues than that in normal gastric mucosa tissues (Figure 1(a)). The overall survival of gastric cancer patients with low expression of UBAP2L was better than those with high expression (Figure 1(b)).

a. The expression levels of UBAP2L in gastric cancer and normal gastric mucosa tissues. b. The overall survival of gastric cancer patients with high or low UBAP2L expression levels. (*p < 0.05)

**Figure 2.**
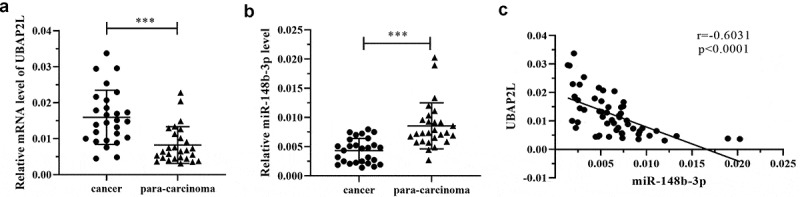
The expression of UBAP2L was increased in gastric cancer tissues

The expression of UBAP2L and miR-148b-3p was detected in 27 pairs of gastric cancer and para-carcinoma normal gastric mucosa tissues by real-time PCR. The data revealed that UBAP2L expression was upregulated and miR-148b-3p expression was downregulated in gastric cancer specimens (Figure 2(a,b)). Correlation analysis revealed that the expression of UBAP2L was negatively correlated with that of miR-148b-3p in gastric cancer tissues (Figure 2(c)).

a. The mRNA levels of UBAP2L in twenty-seven paired of gastric cancer and para-carcinoma gastric mucosa tissues were measured by real-time PCR. b. The level of miR-148b-3p in gastric cancer and paired para-carcinoma gastric mucosa samples were determined by real-time PCR. c. The correlation between the expressions of UBAP2L and miR-148b-3p was analyzed using the Spearman test. (***p < 0.001)

### 3.2. UBAP2L promoted proliferation and repressed apoptosis of gastric cancer cells

**Figure 3.**
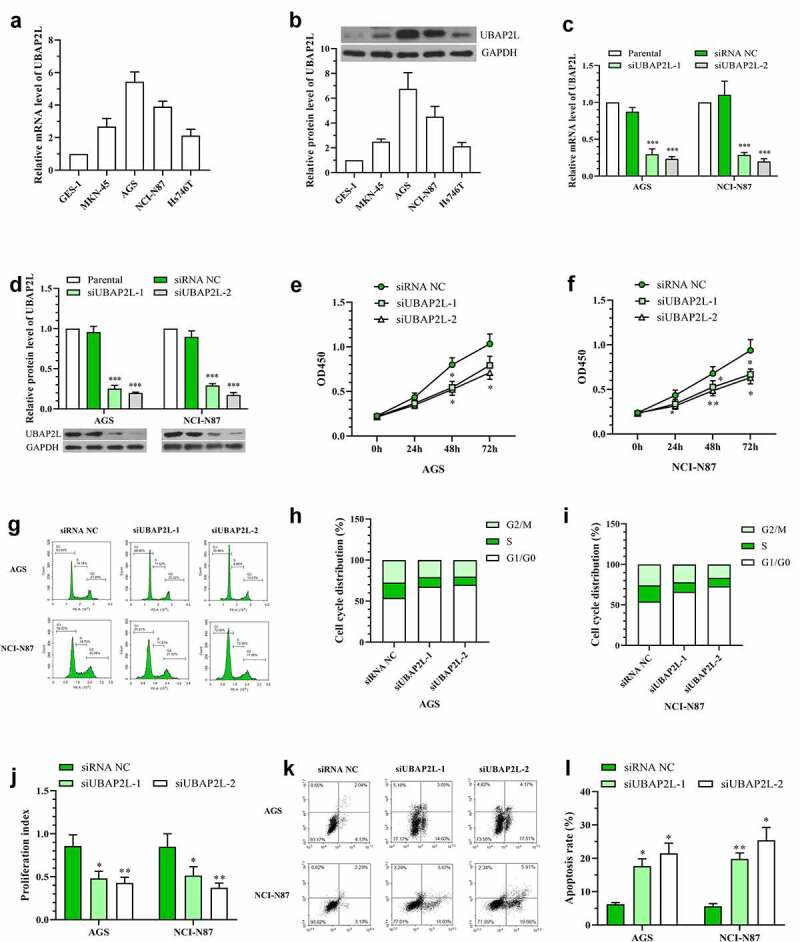
Silencing of UBAP2L repressed proliferation and promoted apoptosis of gastric cancer cells

Next, the expression levels of UBAP2L were determined in normal human gastric mucosa cell GES-1 and four human gastric cancer cell lines AGS, MKN45, Hs726T, and NCI-N87. As shown in Figure 3(a,b), UBAP2L was highly expressed in gastric cancer cells compared with that in normal gastric mucosa cells.

a and b. The mRNA and protein levels of UBAP2L in normal human gastric mucosa cells and gastric cancer cells were determined by real-time PCR and western blot, respectively. c and d. The mRNA and protein levels of UBAP2L in AGS and NCI-N87 cells were detected after transfection of siRNA targeting UBAP2L. e and f. CCK-8 assay was performed for measurement of cell viability in UBAP2L-silenced AGS and NCI-N87 cells. g-i. The cell cycle distribution of AGS and NCI-N87 cells was examined by flow cytometry. j. The proliferation index (PI) of AGS and NCI-N87 cells were calculated based on the cell numbers in each phase of the cell cycle (PI = (cell numbers in G2/M and S phases)/cell numbers in G1/G0 phase). k and l. The apoptosis of AGS and NCI-N87 cells were detected by flow cytometry. (*p < 0.05, **p < 0.01, ***p < 0.001, compared with siRNA NC)

In order to investigate the effect of UBAP2L on the biological behaviors of gastric cancer cells, UBAP2L was silenced in AGS and NCI-N87 cells, and overexpressed in MKN-45 and Hs746T cells. The transfection effectiveness was validated by real-time PCR and western blot analyses. The mRNA and protein expression of UBAP2L were decreased by more than 70% in cells transfected with siRNA targeting UBAP2L (Figure 3(c,d)), and increased by more than 5 folds after administration of overexpression plasmid (Fig. S1a and S1b). Subsequently, CCK-8 assay showed a significant decline in the viability of UBAP2L-silenced NCI-N87 and AGS gastric cells (Figures 3(e,f)). Flow cytometry results revealed that knockdown of UBAP2L delayed G1/G0 to S phase transition (Figure 3(g,j)) and induced obvious apoptosis (Figures 3(k,l)) in both NCI-N87 and AGS cells. On the contrary, the forced expression of UBAP2L promoted viability, cell cycle transition, and suppressed apoptosis in MKN-45 and Hs746T cells (Fig. S1c-i).

### 3.3. UBAP2L facilitated migration and invasion of gastric cancer cells

**Figure 4.**
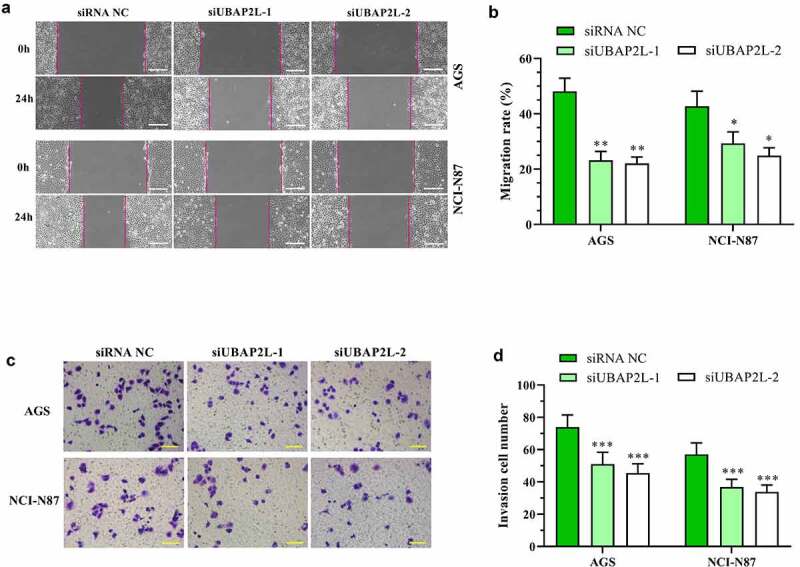
Knockdown of UBAP2L suppressed migration and invasion of gastric cancer cells

The effect of UBAP2L on cell migratory and invasive ability was examined by wound healing and transwell assays, respectively. As shown in Figure 4(a,b), the migration of the AGS and NCI-N87 cells was significantly reduced by silencing of UBAP2L. Similarly, cell invasion was decreased after the UBAP2L knockdown (Figure 4(c,d)). Gain-of-function experiments displayed that the overexpression of UBAP2L facilitated migratory and invasive ability of MKN-45 and Hs746 cells (Fig. S2a-d).

a and b. Wound healing assays were performed to measure the migratory ability of AGS and NCI-N87 cells after knockdown of UBAP2L. (the scale bar represented 200 μm) c and d. Transwell assay was used for detection of AGS and NCI-N87 cell invasion. (the scale bar represented 100 μm) (*p < 0.05, **p < 0.01, ***p < 0.001, compared with siRNA NC)

### 3.4. UBAP2L functioned by regulating Wnt/β-catenin signaling

**Figure 5.**
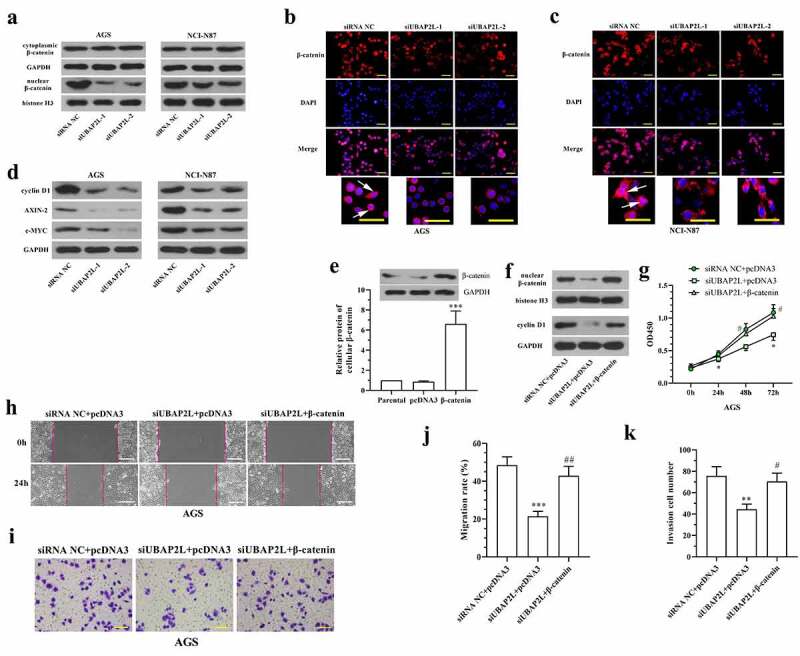
UBAP2L functions by regulating Wnt/β-catenin signaling

In consideration of the important role of Wnt/β-catenin signaling in tumorigenesis, the influence of UBAP2L on this signaling was assessed. Western blot and immunofluorescent staining results revealed that the nuclear level of β-catenin was reduced in UBAP2L-silenced gastric cancer cells (Figure 5(a,c)). The expression levels of the downstream target genes (cyclin D1, AXIN-2, and c-MYC) of Wnt/β-catenin signaling were decreased by depletion of UBAP2L (Figure 5(d)). Conversely, the overexpression of UBAP2L induced an increased level of nuclear β-catenin (Fig. S2e) and promoted expression of cyclin D1, AXIN-2, and c-MYC in MKN-45 and Hs746T cells (Fig. S2f). From these results, we demonstrated that UBAP2L induced activation of Wnt/β-catenin signaling in gastric cancer cells.

a. The levels of β-catenin in cytoplasm and nuclei were analyzed by western blot after knockdown of UBAP2L. b and c. The intracellular distribution of β-catenin was observed through immunofluorescence staining. (the scale bar represented 50 μm, arrows indicated nuclear location of β-catenin) d. The expression levels of several Wnt/β-catenin targets, including cyclin D1, AXIN-2, and c-MYC, were examined by western blot. e. The cellular level of β-catenin was detected after overexpression of β-catenin. f. The levels of nuclear β-catenin and cellular cyclin D1 were detected in AGS cells after knockdown of UBAP2L and/or overexpression of β-catenin. g. Cell viability was measured by CCK-8 assay. h-k. Cell migration and invasion were detected by wound healing and transwell assays, respectively. (the scale bar represented 200 μm in h, and represented 100 μm in i) (*p < 0.05, **p < 0.01, ***p < 0.001, compared with pcDNA3 or siRNA NC+pcDNA3, #p < 0.05, ##p < 0.01, compared with siUBAP2L+pcDNA3)

We further investigated the role of Wnt/β-catenin signaling in the regulation of UBAP2L. The continuously active β-catenin, overexpression plasmid was generated from wild-type β-catenin with mutation of Ser33 to Tyr in pcDNA3.1 vector, which was transfected into UBAP2L-silenced AGS cells. After transfection, the ectopic expression of β-catenin in AGS cells was confirmed by western blot (Figure 5(e)). The accumulation of nuclear β-catenin and increased expression of the cyclin D1 indicated the activation of Wnt/β-catenin signaling in AGS cells after overexpression of β-catenin (Figure 5(f)). CCK-8 assay showed that the inhibition of cell proliferation induced by UBAP2L knockdown was abolished by enhanced expression of β-catenin (Figure 5(g)). The mobility of UBAP2L-silenced cells was enhanced by additional activation of β-catenin (Figure 5(h,k)). The results demonstrated that the anti-tumor effect of UBAP2L knockdown was antagonized by overexpression of β-catenin, suggesting that silencing of UBAP2L may restrain proliferation, migration, and invasion of gastric cancer cells by deactivating Wnt/β-catenin signaling.

### 3.5. Knockdown of UBAP2L restrained the tumorigenesis of gastric cancer cells in nude mice

**Figure 6.**
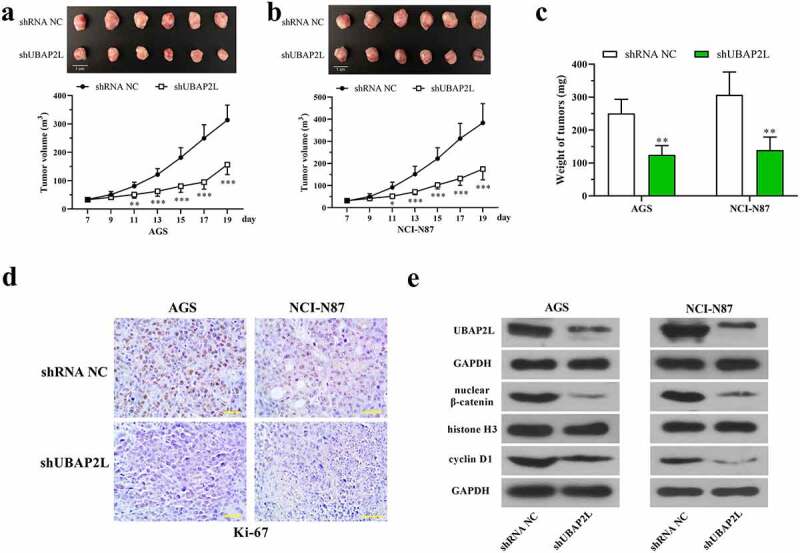
Knockdown of UBAP2L restrained the tumorigenesis of gastric cancer cells in nude mice

To confirm the role of UBAP2L *in vivo*, the stably UBAP2L-silenced AGS, and NCI-N87 cells were subcutaneously inoculated into nude mice, and the subcutaneous tumors were isolated at day 19. Figure 6(a,b) displayed that the volume of tumors was significantly reduced by silencing of UBAP2L. Similarly, the weight of tumors formed by UBAP2L-knocked down cells was also increased. Immunohistochemical staining showed a reduction in the expression of Ki-67 in UBAP2L-silence tumors (Figure 6(d)). Western blots revealed a decreased level of nuclear β-catenin and cellular cyclin D1 accompanied by a downregulation of UBAP2L in tumors (Figure 6(e)), suggesting that UBAP2L knockdown inhibited wnt/β-catenin signaling *in viv*o.

The stably UBAP2L-silenced AGS and NCI-N87 cells or control cells were inoculated in nude mice, and the subcutaneous nodules were isolated after 19 days. a and b. The images and volume of tumors. c. The weight of tumors. d. Immunohistochemical staining was performed to determine the expression of Ki-67 in subcutaneous tumors. (the scale bar represented 50 μm) e. The levels of nuclear β-catenin and cellular UBAP2L and cyclin D1 are found in tumors. (*p < 0.05, **p < 0.01, ***p < 0.001, compared with shRNA NC)

### 3.6. UBAP2L was downregulated by miR-148b-3p in gastric cancer cells

**Figure 7.**
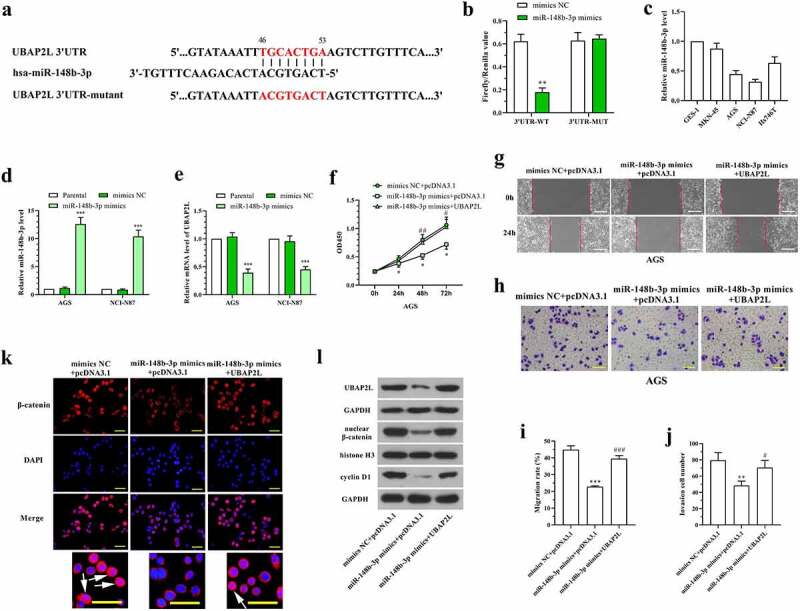
UBAP2L was downregulated by miR-148b-3p in gastric cancer cells

The database TargetScan was used for bioinformatic prediction of UBAP2L upstream miRNAs. As shown in Figure 7(a), there are potential binding sites of miR-148b-3p in 3ʹUTR of UBAP2L. Dual-luciferase reporter assay was performed to confirm the binding between miR-148b-3p and UBAP2L. The data showed a reduced luciferase activity in cells cotransfected with pmirGLO vector containing UBAP2L 3ʹUTR sequence and miR-148b-3p mimics (Figure 7(b)). The levels of miR-148-3p were lower in AGS and NCI-N87 cells than that in normal human gastric mucosa cells and other human gastric cancer cells (Figure 7(c)), which was opposite with the expression of UBAP2L. To investigate the effect of miR-148b-3p and its regulation on UBAP2L in gastric cancer cells, miR-148b-3p mimics were transfected into AGS and NCI-N87 cells. The expression level of UBAP2L was decreased significantly by overexpression of miR-148b-3p in gastric cancer cells (Figures 7(d,e)). CCK-8, wound healing, and transwell assays showed that miR-148b-3p-induced alterations of proliferation, migration, and invasion were abolished by enhanced expression of UBAP2L in AGS cells (Figure 7(f,j)). Immunofluorescent staining demonstrated that miR-148b-3p blocked β-catenin entering into nuclei (Figure 7(k)). Western blot analysis revealed that nuclear β-catenin and cellular cyclin D1 levels were reduced by miR-148b-3p (Figure 7(l)). In contrast, the additional overexpression of UBAP2L abolished the inhibitory effect on Wnt/β-catenin signaling activity mediated by miR-148b-3p (Figures 7(k,l)). These results suggested that miR-148b-3p inhibited gastric cancer cell proliferation, migration, and invasion by downregulating UBAP2L expression.

a. The binding sites of miR-148b-3p in the 3ʹUTR of UBAP2L. b. Dual-luciferase reporter assay was performed to confirm the binding between UBAP2L and miR-148b-3p in 293 T cells. c. miR-148-3p levels in human normal gastric mucosa cells and gastric cancer cells were examined using real-time PCR. d and e. Real-time PCR analysis of miR-148b-3p and UBAP2L levels in AGS cells after transfection of miR-148-3p mimics. f. The viability of AGS cells with overexpression of miR-148b-3p and UBAP2L. g-j. Wound healing and transwell assays were performed to measure the migration and invasion in AGS cells, respectively. (The scale bar represented 200 μm in g, and represented 100 μm in h) k. Immunofluorescent staining showing β-catenin distribution in AGS cells. (The scale bar represented 50 μm, arrows indicated nuclear location of β-catenin) l. The levels of nuclear β-catenin and cellular UBAP2L and cyclin D1 in AGS cells. (*p < 0.05, **p < 0.01, ***p < 0.001, compared with mimics NC or mimics NC+pcDNA3.1; #p < 0.05, ##p < 0.01, ###p < 0.001, compared with miR-148b-3p mimcs+pcDNA3.1)

## 4. Discussion

From the data in this study, we deduced that UBAP2L was required by gastric cancer cells to maintain their malignant behaviors, which involved the activation of Wnt/β-catenin signaling pathway. UBAP2L is an ubiquitin-associated protein, and it has been demonstrated to aggregate after treatment of proteasome inhibitor [12]. The ubiquitin-proteasome system (UPS) is in charge of protein degradation in eukaryotes and it is essential for cellular protein homeostasis [29]. Existing reports have proved that 80% of the proteins are degraded via UPS in cells, including those involved in DNA replication and transcription, cell proliferation and senescence, material transportation, angiogenesis, and drug resistance [30–33]. The UPS contains a series of essential components: ubiquitin, E1 ubiquitin-activating enzyme, E2 ubiquitin-conjugating enzyme, E3 ubiquitin ligases, deubiquitinating enzyme (DUB), and 26S proteasome [34]. The proteins for degradation are tagged by polyubiquitination mediated by E1, E2, and E3, and the polyubiquitinated proteins are proteolytic degraded by proteasome complex [35]. There is a higher efficiency of protein turnover in cancer cells, as well as a larger requirement for protein homeostasis compared with normal cells. A functional UPS system is necessary for tumor initiation and development. Cancer cells rely on a high speed of protein turnover. Inhibiting the activities of different components of the UPS has emerged as a potential therapeutic strategy for cancer treatment [36]. For instance, Bortezomib and Ixazomib, targeting 20S core particles of the proteasome, have been approved by the American Food and Drug Administration (FDA) for clinical treatment of cancers [37,38]. UBAP2L has been reported to be overexpressed in multiple cancers, and knockdown of UBAP2L could suppress malignant behaviors of cancer cells. In our study, UBAP2L was demonstrated to be upregulated in gastric cancer tissues, and its overexpression facilitated cell proliferation, migration, invasion, and tumorigenesis in gastric cancer cells. These results suggest that UBAP2L may play a tumor-promoting role in gastric cancer. In addition, UBAP2L activated Wnt/β-catenin signaling, and the persistent activation of β-catenin partly reversed the inhibitory effect of UBAP2L knockdown on malignant behaviors of gastric cancer cells.

Wnt/β-catenin signaling, also named canonical Wnt signaling, is a deeply conserved pathway. It is involved in a wide variety of disorders associated with abnormal cell proliferation in mammals, including malignant tumors. In the absence of Wnt ligands, β-catenin is recruited into a destruction complex that contains adenomatous polyposis coli (APC), glycogen synthase kinase 3 beta (GSK3β), AXIN and casein kinase 1 (CK1), and phosphorylated by CK1 and GSK3β. This leads to the ubiquitination and degradation of β-catenin by USP. When the Wnt ligands bind to membrane receptors, GSK3β is phosphorylated, and the destruction complex degrades, resulting in accumulation of β-catenin in the cytoplasm and thus translocation into the nucleus. Consequently, β-catenin binds to the T-cell factor (TCF)/lymphoid enhancement factor (LEF), and promotes transcription of target genes, such as *cyclin d1, c-myc,* and *axin-2* [39–41]. Mutation at β-catenin Ser33 blocks the phosphorylation and degradation of β-catenin, which leads to persistent activation of Wnt/β-catenin signaling [42]. Abnormal activation of Wnt receptors triggers tumorigenesis and metastasis [43–46]. The data from databases and articles showed high expression of β-catenin in gastric cancer at transcription and translation levels [47,48]. This signaling is considered a therapeutic target for gastric cancer treatment [49,50]. Our study shows that the accumulation of nuclear β-catenin promoted proliferation, migration, and invasion of gastric cancer cells. The tumor-suppressing effect of the UBAP2L knockdown was abolished by β-catenin activation. The results suggest that UBAP2L likely exerts its function in gastric cancer cells by orchestrating Wnt/β-catenin signaling. Moreover, the analysis from database showed the positive correlation between expression of UBAP2L and that of wnt/β-catenin downstream target genes, *CCND1, AXIN2,* and *MYC* in gastric cancer tissues (Fig. S3), suggesting that UBAP2L activated this signaling. However, the detailed mechanism remains unclear. It has been reported that UBAP2L interacted with BMI1 to regulate the progenitor activity of hematopoietic stem cells [51], and BMI1 activated wnt/β-catenin signaling by negatively regulating the WNT antagonist IDAX [52]. Therefore, we hypothesized that UBAP2L may activate wnt/β-catenin signaling by interacting with BMI1. This hypothesis needs to be confirmed by more experiments. On the other hand, UBAP2L is an ubiquitin-associated protein, and we guessed that it may participate in ubiquitination of β-catenin. However, there is no enough evidence to elucidate the detailed functionality of UBAP2L in ubiquitination. Therefore, many experiments are needed to explain, however, UBAP2L activated wnt/β-catenin signaling in the future.

Additionally, the overexpression of β-catenin could not completely reverse the influence of the UBAP2L knockdown in gastric cancer cells, suggesting that β-catenin was not the sole pathway that mediated the function of the UBAP2L. Thus, we demonstrate that UBAP2L may exert tumor-promoting function partly through Wnt/β-catenin signaling in gastric cancer.

In addition, UBAP2L was predicted to be a target of miR-148b-3p. MiR-148b-3p exhibited anti-gastric cancer properties *in vivo* and *in vitro* [16,18]. We demonstrated that miR-148b-3p bound to the 3ʹUTR of UBAP2L mRNA to downregulate its expression. The inhibitory effect of miR-148b-3p on gastric cancer cell proliferation, migration, and invasion was neutralized by the overexpression of UBAP2L to some degree, which suggested that miR-148b-3p functioned, at least in part, by regulating UBAP2L. So far, the tumor-suppressing role of miR-148-3p has been reported broadly in cells and animals, but its clinical value is unknown. In the future, miRNA mimics may be developed as a potential molecular marker or therapy for cancer patients. In a recent paper [53], Jafari *et al.,* found that the effect of transient transfection of miR-34a mimics on target gene repression and cell phenotype was not consistent in gastric cancer cells, and it depended on the concentration of miRNA mimics and cell types. At the same time, the stable transfection of pre-mir-34a efficiently suppressed the target gene expression and altered phenotype of gastric cancer cells. Based on these findings, Jafari *et al*. put forward that the use of pre-miRNA was more reliable and efficient. Meanwhile, the regulatory action of miRNA mimics has been demonstrated by countless studies [54]. What is more, the stable expression of pre-miRNA is difficult to achieve in clinic. Therefore, there is a long-standing dispute regarding it. Anyhow, exciting studies of miRNA still provide potential strategies for clinical treatment of tumors. Of course, many experiments need to be done to confirm its safety and availability.

## 5. Conclusions

We demonstrate that UBAP2L is highly expressed in gastric cancer specimens and cell lines, and its overexpression aggravates the growth and metastasis of gastric cancer cells by activating Wnt/β-catenin signaling. UBAP2L was identified as a target of miR-148b-3p, and their expression levels were negatively correlated in gastric cancer tissues. The anti-gastric tumor effect of miR-148b-3p is possibly related to UBAP2L downregulation. We propose UBAP2L as a potential target for therapeutic treatments against gastric cancer.

## Supplementary Material

Supplemental Material

## Data Availability

All data generated or analyzed during this study are included in this article.
