# Supplementary material for: Ubiquitin-associated protein 2 like (UBAP2L) enhances growth and metastasis of gastric cancer cells
Source: Bioengineered. 2021 Nov 25;12(2):10232–45. doi: 10.1080/21655979.2021.1982308 (PMC8809994; doi:10.1080/21655979.2021.1982308)
Supplement: Supplemental Material [file KBIE_A_1982308_SM6137.zip › supplementary/Figure legends.docx]

**Figure S1. UBAP2L promoted proliferation and inhibited apoptosis of gastric cancer cells**

a and b. Real-time PCR and western blot were used to confirm the effectiveness of UBAP2L overexpression plasmid. c and d. The viability of MKN-45 and Hs746T cells were determined by CCK-8 assay. e and f. The distribution of cells in each phase of cell cycle was measured by flow cytometry. g. The proliferation index (PI) was calculated according to data in e and f. h and i. The apoptosis rates of MKN -45 and Hs746T cells were detected by flow cytometry. (**p<0.05, **p<0.01, ***p<0.001 compared with pcDNA3.1; PI=(G1/G0+S)G2/M*)

**Figure S2. UBAP2L enhanced migration and invasion of gastric cancer cells.**

a and b. Wound healing assay was performed to measure migratory ability of MKN-45 and Hs746T cells (the scale bar represented 200 μm). c and d. Transwell assay was carried out to examine invasive ability of MKN-45 and Hs746T cells (the scale bar represented 100 μm). e. The cytoplasmic and nuclear levels of β-catenin in MKN-45 and Hs746T cells after enhanced expression of UBAP2L. f. The protein levels of cyclin D1, AXIN-2 and c-MYC in MKN-45 and Hs746T cells after ectopic expression of UBAP2L. (**p<0.05, **p<0.01 compared with pcDNA3.1*)

**Figure S3. The expression level of UBAP2L was positively correlated with that of cyclin D1, AXIN-2 and c-MYC in gastric cancer tissues.**

The correlation between expression of UBAP2L and that of cyclin D1 (a), AXIN-2 (b) or c-MYC (c) was analyzed by GEPIA database.
