# Supplementary figures and images for: Ubiquitin-associated protein 2 like (UBAP2L) enhances growth and metastasis of gastric cancer cells
Source: Bioengineered. 2021 Nov 25;12(2):10232–45. doi: 10.1080/21655979.2021.1982308 (PMC8809994; doi:10.1080/21655979.2021.1982308)

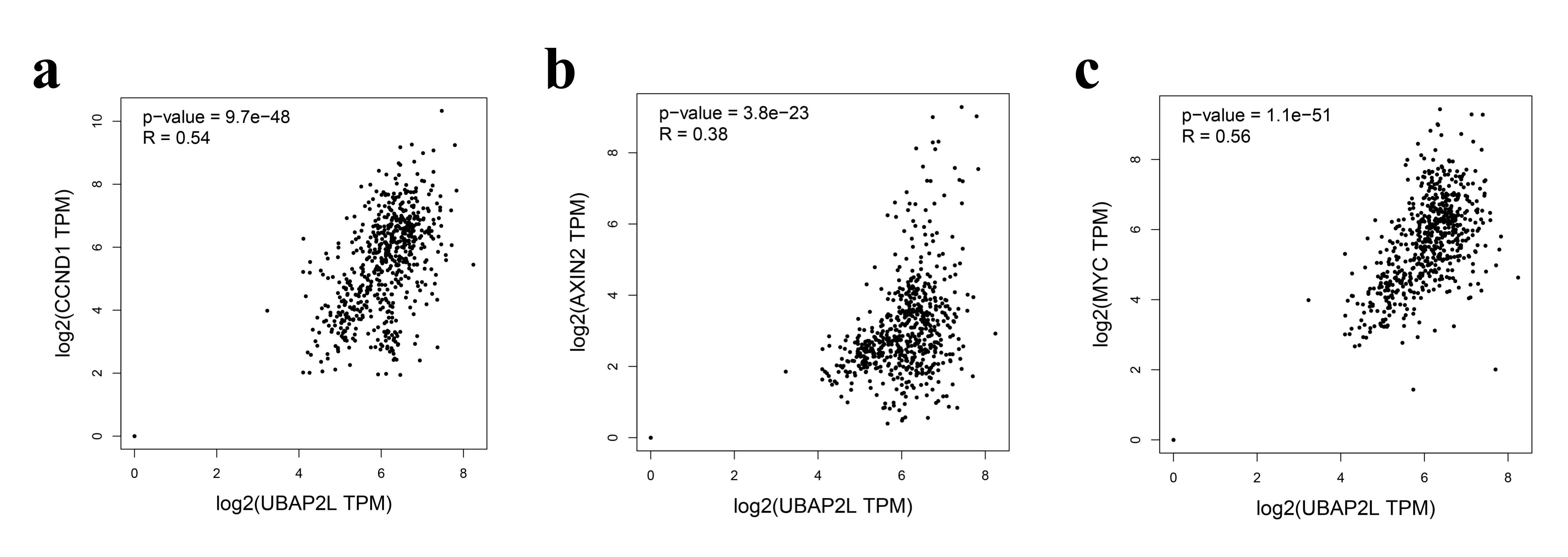

Supplement: Supplemental Material [file KBIE_A_1982308_SM6137.zip › supplementary/figrue S3.jpg]

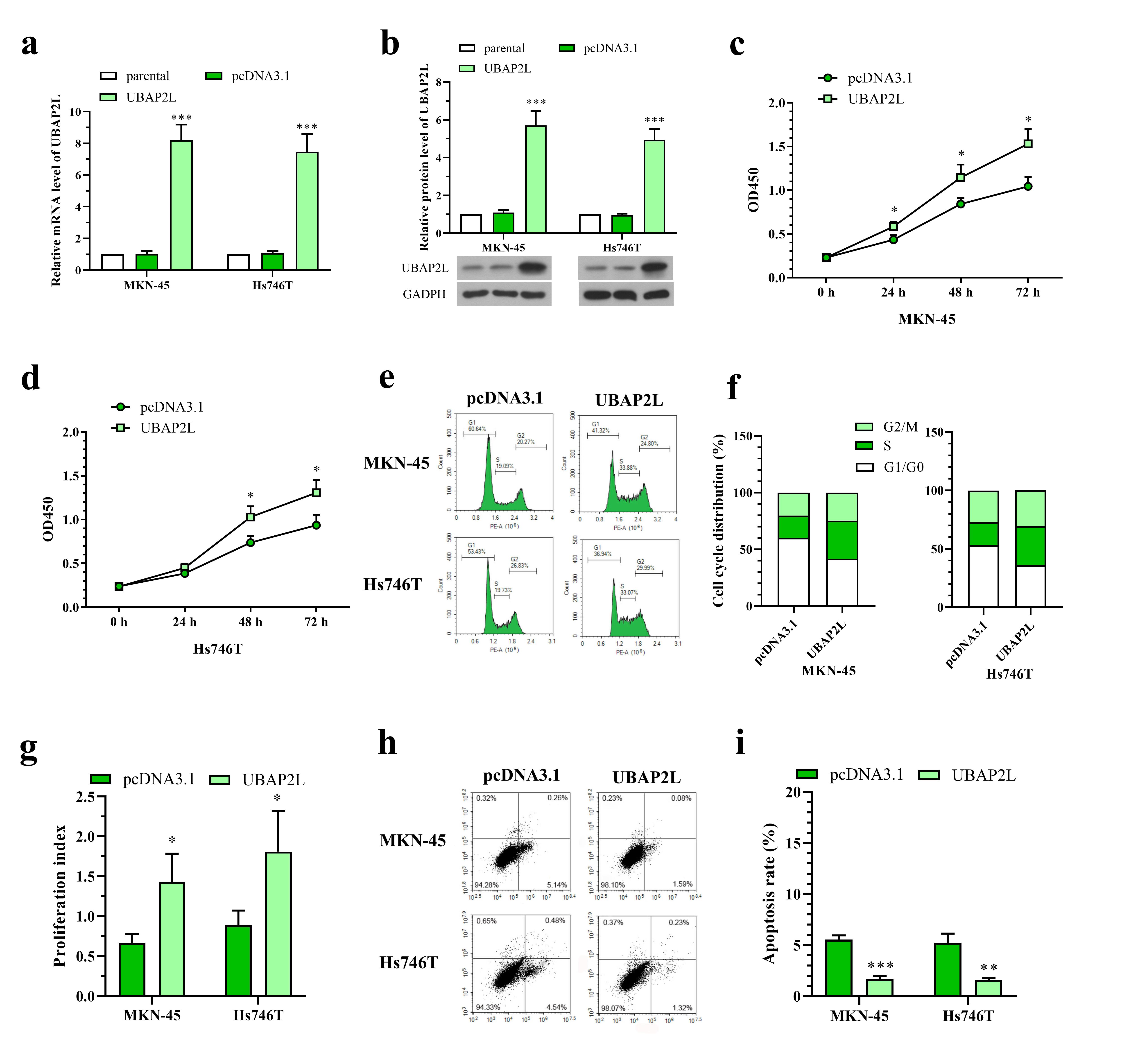

Supplement: Supplemental Material [file KBIE_A_1982308_SM6137.zip › supplementary/figure S1.jpg]

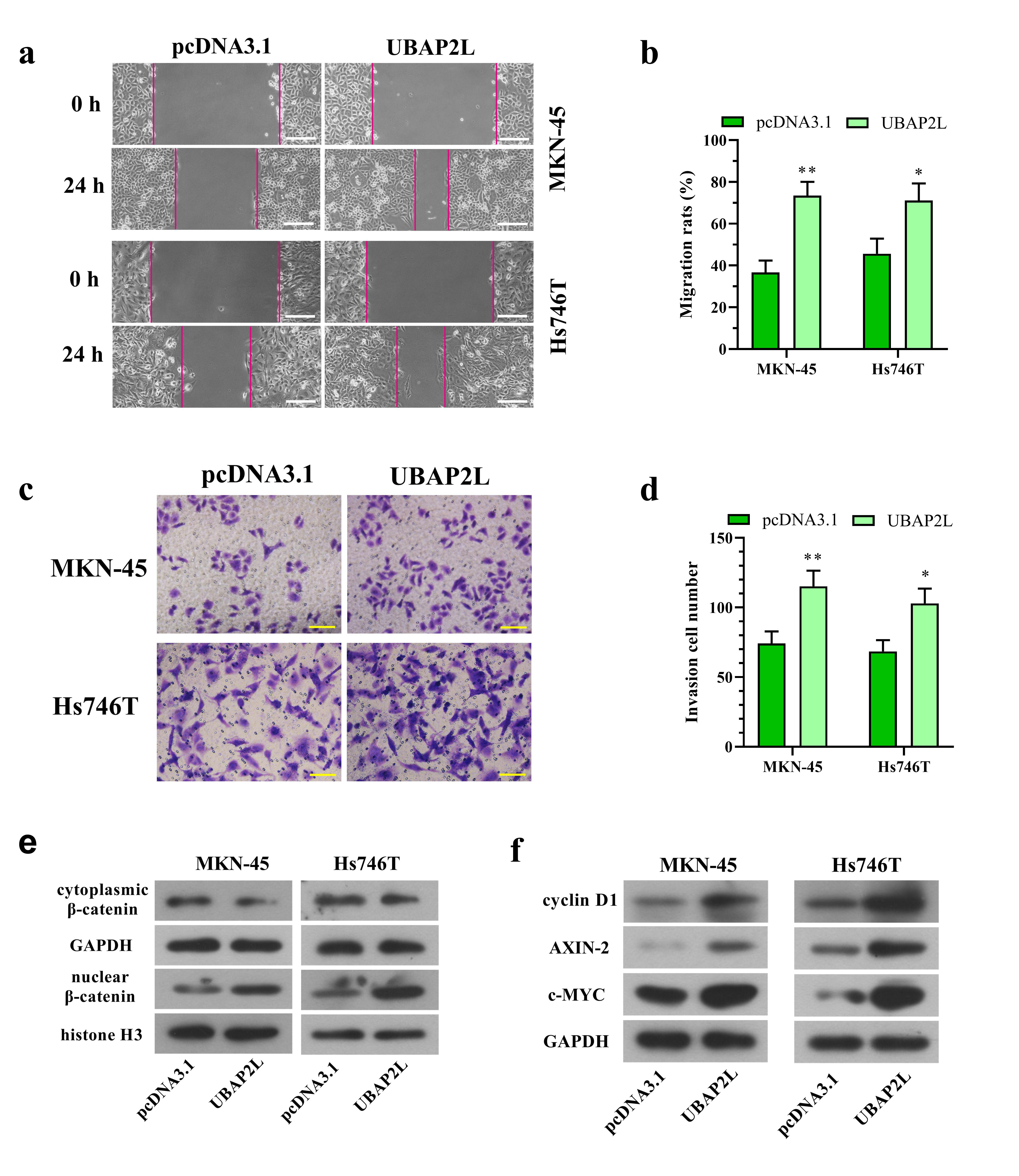

Supplement: Supplemental Material [file KBIE_A_1982308_SM6137.zip › supplementary/figure S2.jpg]
